# Computed Tomographic Angiography and Yield for Gastrointestinal Bleeding in the Emergency Department

**DOI:** 10.1001/jamanetworkopen.2025.29746

**Published:** 2025-08-29

**Authors:** Siona Prasad, C. Michael Hood, Cameron Young, Joshua A. Hirsch, Sanjay Saini, Aaron D. Sodickson, Michael H. Lev, Ali S. Raja, Michael S. Gee, David C. Whitehead, Marc D. Succi

**Affiliations:** 1 Medically Engineered Solutions in Healthcare Incubator, Innovation in Operations Research Center (MESH IO), Mass General Brigham, Boston, Massachusetts; 2 Harvard Medical School, Boston, Massachusetts; 3 Department of Radiology, Massachusetts General Hospital, Boston; 4 Department of Radiology, Brigham and Women’s Hospital, Boston, Massachusetts; 5 Enterprise Radiology, Mass General Brigham, Boston, Massachusetts; 6 Department of Emergency Medicine, Massachusetts General Hospital, Boston

## Abstract

**Importance:**

Overuse of advanced imaging in the emergency department (ED) contributes to higher costs, reduced efficiency, and crowding. Computed tomographic angiography (CTA) is a recommended first-line diagnostic for acute gastrointestinal bleeding (GIB), yet its increasing use may not always improve detection of active bleeding.

**Objective:**

To evaluate recent trends in CTA use for suspected GIB in the ED and assess changes in diagnostic yield.

**Design, Setting, and Participants:**

This retrospective cohort study included all adult patients who underwent CTA of the abdomen and pelvis for suspected GIB at a 1011-bed urban academic medical center between January 2017 and December 2023.

**Exposure:**

Suspected GIB prompting a CTA order in the ED.

**Main Outcomes and Measures:**

The primary outcome was the annual number and proportion of GIB-related CTAs among all ED computed tomography (CT) examinations. The secondary outcome was the test-positive proportion (diagnostic yield), defined as the percentage of CTAs showing active bleeding or evidence of hemorrhage. All CTAs were interpreted by board-certified emergency radiologists and reviewed by a fellowship-trained emergency radiologist.

**Results:**

Among 954 ED patients (mean [SD] age, 66.7 [6.3] years; 427 female [44.8%]), the number of GIB-related CTA examinations increased from 30 of 32 197 ED CT examinations (0.09%) in 2017 to 288 of 44 423 (0.65%) in 2023. Over the same period, the test-positive proportion declined from 6 of 30 (20.0%) in 2017 to 18 of 288 (6.3%) in 2023. Multivariable analysis showed that more recent calendar year was associated with lower odds of a test-positive examination (OR, 0.84; 95% CI, 0.73-0.96; *P* = .01), older age with higher odds (OR, 1.02; 95% CI, 1.00-1.04; *P* = .02), and active cancer with lower odds (OR, 0.35; 95% CI, 0.12-1.00; *P* = .05).

**Conclusions and Relevance:**

CTA use increased substantially over 7 years while diagnostic yield declined. This trend highlights the need to balance the diagnostic benefit of CTA with interpretation time, radiation exposure, and operational strain. These findings support a need for evidence-based ordering criteria and decision-support tools to help guide CTA use in the ED evaluation of gastrointestinal bleeding.

## Key Points

**Question:**

How has use of computed tomographic angiography (CTA) for suspected gastrointestinal bleeding in the emergency department (ED) increased, and has it led to improved diagnostic detection or possible overuse?

**Findings:**

In this retrospective cohort study of 954 ED patients from 2017 to 2023, the proportion of total CTA examinations related to gastrointestinal bleeding increased from 0.09% to 0.7%. The test-positive proportion declined from 20.0% to 6.3%.

**Meaning:**

These findings suggest potential overuse of CTA in the ED setting for suspected gastrointestinal bleeding.

## Teaser

This cohort study examines recent trends in the use of computed tomographic angiography for suspected gastrointestinal bleeding in the emergency department and estimates changes in diagnostic yield at an urban academic medical center.

## Introduction

Gastrointestinal bleeding (GIB) is a common and potentially life-threatening condition encountered in emergency department (ED) settings, contributing to high morbidity, mortality, and health care resource consumption, remaining the leading cause of gastrointestinal–related hospitalizations in the US.^1,2^ Prompt, accurate evaluation of suspected GIB in the ED is therefore essential for improving outcomes and reducing costs.

Computed tomographic angiography (CTA) has become a principal diagnostic tool for suspected GIB, providing high sensitivity (85% to 90%), specificity (approximately 92%), and accuracy (approximately 95%).^3^ Its robust negative estimative value is associated with reduced rebleeding and lower intervention rates, supporting its use as a potential means to decrease hospital admissions.^4,5^ Accordingly, joint guidelines from the American College of Gastroenterology and Society of Abdominal Radiology (ACG/SAR) endorse CTA as first-line imaging for hemodynamically unstable patients and select hemodynamically stable patients with strong suspicion for active GIB.^6,7^ However, CTA’s utility presents tradeoffs—CTA is a multiphase examination that requires the radiologist to review a relatively high number of images, and findings of active bleeding can be subtle, increasing interpretive complexity and radiologist workload.^8^ These factors may exacerbate ED crowding and delay patient throughput. Rising imaging volumes in ED settings further impact workflow and resource allocation.^9,10,11,12,13,14,15^

While CTA is well established in diagnosing and guiding treatment in patients with clear evidence of GIB,^16,17^ increased use of CTA in the ED may be driven by nonguideline-based practice, defensive medicine, or heightened patient expectations. Few studies have examined longitudinal trends in CTA ordering for suspected GIB or assessed changes in test-positive proportion (ie, diagnostic yield) over time. Determining whether higher CTA use increases GIB detection or reflects potential overuse is essential for imaging stewardship.

This retrospective cohort study evaluated 7-year trends in CTA use for suspected acute GIB in the ED of a large academic medical center, quantified changes in test-positive proportion, and assessed shifts in patient and examination characteristics. These findings may inform evidence-based imaging criteria and promote judicious CTA use in the evaluation of acute gastrointestinal bleeding.

## Methods

### Design, Setting, and Participants

This retrospective observational cohort study was conducted at a 1011-bed quaternary care urban academic medical center with a 66-bed ED evaluating approximately 110 000 patient visits annually. All adult patients (aged 18 years or older) who underwent CTA of the abdomen and/or pelvis for suspected acute GIB between January 1, 2017, and December 31, 2023, were eligible. Examinations performed for indications unrelated to GIB were excluded.

**Figure 1.**
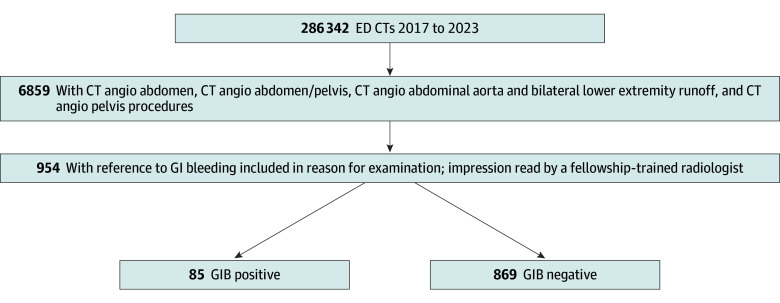
Patient Inclusion and Study Flow Diagram Flow diagram illustrates selection of eligible patients undergoing computed tomography angiography for suspected gastrointestinal bleeding in the emergency department. CT indicates computed tomography; ED, emergency department; GIB, gastrointestinal bleeding.

Eligible patients were identified by electronic query of the institutional radiology information system using examination codes for abdominal and pelvic CTA, further filtered by clinician-documented reason for examination containing terms such as gastrointestinal bleed or equivalent. Details of this selection process are summarized in Figure 1.

This study was approved by the institutional review board with a waiver of informed consent, in accordance with the Health Insurance Portability and Accountability Act (HIPAA). Because data collection used retrospective chart review, the requirement for informed consent was waived. All patient health information was protected through secure data handling and deidentification procedures. The study was reported in compliance with the Strengthening the Reporting of Observational Studies in Epidemiology (STROBE) reporting guideline.^18^

### Study Variables

Primary variables included total ED CT examination volume, volume of GIB-related CTA examinations, and the test-positive proportion. The primary outcome was annual GIB-related CTA examination volume, expressed as an absolute count and as a proportion of all ED CT examinations per year. The secondary outcome was the test-positive proportion of CTA examinations, defined as the number of GIB-related CTAs demonstrating imaging evidence of active bleeding or evidence of hemorrhage (including arterial or delayed phases) divided by the total number of GIB-related CTA examinations.

All CTA examinations were initially interpreted by board-certified emergency radiologists, and each report was subsequently reviewed by a fellowship-trained emergency radiologist to classify findings as test-positive or test-negative for GIB. Demographic variables (age, sex, race, and presence of active cancer) were collected for all patients who received GIB-related CTA examinations. Race was patient-reported and obtained from the electronic health record. Categories included Asian, Black, White, and Other or unknown. Other or unknown included American Indian, Alaska Native, Pacific Islander, individuals who did not identify with another category, and patients with missing documentation. Race was included as a demographic variable to assess potential subgroup differences. However, it was not statistically significant in any analysis and did not affect the study's primary outcomes.

### Statistical Analysis

For each year, the total number of ED CT examinations, GIB-related CTA examinations, and test-positive GIB-related CTA examinations were calculated. The proportion of GIB-related CTA examinations relative to all ED CT examinations and the test-positive proportion were plotted annually. Least squares linear regression was used to evaluate trends in GIB-related CTA examination volume and test-positive proportion.

Descriptive statistics were used for demographic variables, summarized overall and by test-positive vs test-negative groups. Means (SDs) were calculated for continuous variables and frequencies (No. [%]) for categorical variables. Variables associated with the test-positive proportion in univariable analyses were entered into a multivariable logistic regression model to assess independent associations with a test-positive exam. All statistical tests were 2-tailed, with significance defined as *P* < .05. Analyses were performed using IBM SPSS version 29 (IBM), and results are presented with SDs or 95% CIs where appropriate. Data were analyzed from May to September 2024.

## Results

**Figure 2.**
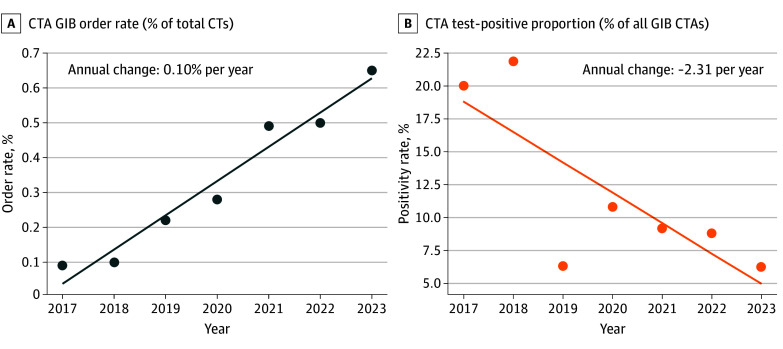
Trends in GIB-Related Computed Tomography Angiography (CTA) Examination Volume and Test-Positive Proportion, 2017 to 2023 Each point reflects annual rates of GIB-related CTA examinations and test-positive proportions from 2017 through 2023. Solid lines represent linear regression models. Slopes denote annual change. CT indicates computed tomography; ED, emergency department; GIB, gastrointestinal bleeding.

Between January 1, 2017, and December 31, 2023, a total of 954 patients (mean [SD] age, 66.7 [6.3] years; 427 female [44.8%]) underwent GIB-related CTA examinations (Table 1). The annual number of GIB-related CTA examinations rose from 30 of 32 197 ED CT examinations (0.09%) in 2017 to 288 of 44 423 (0.7%) in 2023, an annual increase of 0.09% (95% CI, 0.07% to 0.12%; *P* < .001). Over the same period, the test-positive proportion declined from 6 of 30 (20.0%) to 18 of 288 (6.3%) (annual decrease, –1.60%; 95% CI, –2.41% to –0.79%; *P* = .001). Annual trends are shown in Figure 2.

**Table 1.** Annual Volume of GIB-Related CTA Examinations and Test-Positive Proportion in the Emergency Department (2017-2023)

Year	Total ED CTs	No. of GIB CTAs, % of total ED CTs	Positive CTA/total No., %^a^
2017	32 197	30 (0.09)	6/30 (20.0)
2018	32 762	32 (0.1)	7/32 (21.9)
2019	36 106	79 (0.2)	5/79 (6.3)
2020	36 313	102 (0.3)	11/102 (10.8)
2021	42 405	207 (0.5)	19/207 (9.2)
2022	43 244	216 (0.5)	19/216 (8.8)
2023	44 423	288 (0.7)	18/288 (6.3)

Abbreviations: CT, computed tomography; CTA, computed tomography angiography; ED, emergency department; GIB, gastrointestinal bleeding.

^a^ Test-positive proportion refers to the percentage of GIB-related CTA examinations demonstrating active bleeding or other evidence of hemorrhage.

Among the 85 test-positive examinations (8.9%), the mean (SD) age was 71.0 (1.5) years, compared with 66.3 (0.6) years among test-negative examinations (869 [91.1%]). Active cancer was present in 4 (4.7%) vs 87 (10.0%) patients, respectively. Distributions of sex and race are provided in Table 2. In multivariable logistic regression (Table 3), more recent calendar year was associated with lower odds of a test-positive examination (odds ratio [OR], 0.84; 95% CI, 0.73-0.96; *P* = .01), older age with higher odds (OR, 1.02; 95% CI, 1.00-1.04; *P* = .02), and active cancer with lower odds (OR, 0.35; 95% CI, 0.12-1.00; *P* = .05).

**Table 2.** Patient Characteristics and Test Positivity for CTA Examinations Performed for Suspected Gastrointestinal Bleeding

Characteristic	Overall (n = 954)	CTA, No. (%)		*P* value
		Positive (n = 85)^a^	Negative (n = 869)	
Age, mean (SD), y	66.73 (6.33)	71.02 (1.54)	66.29 (0.57)	.008
Sex				
Female	427 (44.8)	36 (42.4)	391 (45.0)	.64
Male				
Cancer	91 (9.54)	4 (4.71)	87 (10.0)	.11
Race				
Asian	60 (6.29)	9 (10.6)	51 (5.87)	.26
Black	86 (9.01)	10 (11.8)	76 (8.75)	
White	679 (71.2)	60 (70.6)	619 (71.2)	
Other or unknown^b^	129 (24.6)	6 (7.1)	123 (14.2)	

Abbreviations: CTA, computed tomography angiography; GIB, gastrointestinal bleeding.

^a^ Test-positive examinations refer to those demonstrating active gastrointestinal bleeding.

^b^ Other or unknown category included American Indian, Alaska Native, Pacific Islander, and patients for whom race was not documented in the medical record.

**Table 3.** Multivariable Logistic Regression of Factors Associated With Test-Positive CTA Examination^a^

Factor	OR (95% CI)	*P* value
Year	0.84 (0.73-0.96)	.01
Age	1.02 (1.00-1.04)	.02
Cancer	0.35 (0.12-1.00)	.05

Abbreviations: CTA, computed tomography angiography; GIB, gastrointestinal bleeding.

^a^ Multivariable logistic regression model includes year of examination, patient age, and active cancer status.

## Discussion

In this cohort study of 954 ED patients who underwent CTA examinations for suspected gastrointestinal bleeding, the proportion of total ED GIB-related CTA examinations rose from 0.09% in 2017 to 0.7% in 2023, while the test-positive proportion declined from 20.0% to 6.3%. In multivariable analysis, older age was associated with higher odds of a test-positive examination, whereas active cancer was associated with lower odds. Sex and race were not independently associated with a test-positive result. These findings suggest that expanding CTA use has been accompanied by diminishing diagnostic yield.

Multiple factors may contribute to increased CTA examination volume for suspected GIB. Although current ACG/SAR guidelines provide direction on appropriate use, clinicians may still order CTA examinations in borderline scenarios when intermittent GIB is suspected as a potential etiology, such as unexplained abdominal pain, drop in hemoglobin, or historical bleeding. Pressures to expedite ED disposition, defensive practice, and increasing patient expectations may further influence ordering behavior.^19,20^ While a test-negative examination can facilitate triage by excluding active bleeding, the declining test-positive proportion in this study may highlight a need for clearer imaging criteria for ordering CTA.

The association between older age and a higher likelihood of a test-positive examination may reflect an increased baseline risk of bleeding among older adults due to greater prevalence of vascular disease, frailty, and more frequent anticoagulant use.^21^ The lower test-positive proportion in patients with active cancer could be influenced by both clinical practice patterns and disease-related factors. For example, ED clinicians may adopt a lower threshold for ordering CTA examinations in patients who have known cancer, especially gastrointestinal. For these patients, CTA can assess for active bleeding, tumor progression, and vascular complications. However, bleeding in patients with malignancy, especially from mucosal or metastatic lesions, is frequently intermittent or slow.^22^ This pattern can reduce the likelihood of detection on a single CTA examination.

### Limitations

This study has limitations. It was conducted at a single urban academic center, which may restrict generalizability to other settings. Tagged red blood cell scintigraphy is a comparator modality to CTA for assessing GIB, but it is not available in our ED. More detailed clinical variables, such as laboratory values, medication use, and the frequency of direct-to-endoscopy or endovascular management, were not available for systematic extraction. Downstream clinical management and patient disposition following each CTA examination were not assessed within the scope of this study, and test-negative examinations may have revealed alternative actionable findings that influenced disposition. Consequently, we do not draw conclusions regarding the necessity of imaging itself. Although all reports were reviewed by a fellowship-trained emergency radiologist, interrater reliability was not formally measured.

Balancing the diagnostic advantages of CTA with concerns about radiation exposure, radiologist workload, and resource use remains a challenge in emergency radiology. Future studies should incorporate more granular clinical data, include outcome assessment after both test-positive and test-negative CTA examinations, and evaluate the impact of decision-support tools and risk-stratification frameworks across multiple centers. Such efforts may help optimize CTA use and ensure that imaging is reserved for clinical contexts where it provides the greatest benefit.

## Conclusions

In a high-volume ED, GIB-related CTA examination volume increased substantially over 7 years, while the proportion of test-positive findings declined. This trend raises considerations about the balance between diagnostic benefit and the demands of advanced imaging, including increased interpretation time, radiation exposure, and system-level strain. These findings support a need for evidence-based criteria and clinical decision-support tools to promote more targeted use of CTA in the ED evaluation of gastrointestinal bleeding.

## Notes

Supplement.
Data Sharing Statement
